# The Impact of Dry Eye Disease on Corneal Biomechanics Analyzed with Corneal Visualization Scheimpflug Technology

**DOI:** 10.3390/biomedicines13102524

**Published:** 2025-10-16

**Authors:** Li-Wen Chiu, Ren-Wen Ho, Hun-Ju Yu, Po-Chiung Fang, I-Hui Yang, Ming-Tse Kuo

**Affiliations:** 1 Department of Ophthalmology, Kaohsiung Chang Gung Memorial Hospital, College of Medicine, Chang Gung University, Kaohsiung City 83301, Taiwan; 2 School of Medicine, Chang Gung University, Taoyuan City 33302, Taiwan; 3 School of Medicine, College of Medicine, National Sun Yat-Sen University, Kaohsiung City 80424, Taiwan

**Keywords:** dry eye disease, dry eye severity, tear film instability, tear film homeostasis, corneal biomechanics

## Abstract

**Background/Objectives**: Dry eye disease (DED) is an ocular surface disease with unstable tear film hemeostasis that could influence the corneal biomechanics. The study aimed to elucidate the impact of dry eye severity on corneal biomechanics. **Methods**: This is a prospective cohort study that enrolled 72 participants with or without dry eye severity. All subjects received dry eye and corneal biomechanic assessment. Dry eye patients were divided into non-DED (>6 s) and DED (<6 s) groups based on the average non-invasive keratograph tear break-up time to compare their performance in corneal biomechanics. We further analyzed the correlation between the corneal biomechanic parameters and dry eye indexes for these patients. **Results**: In this study, 38 non-DED patients and 34 DED patients were enrolled for analysis. The two groups showed significant differences in first applanation (A1) deflection area (*p* = 0.002), A1 delta arc length (*p* = 0.024), second applanation (A2) deformation amplitude (*p* = 0.024), and whole eye movement [mm] (*p* = 0.021). Moreover, both A1 deflection area and A1 delta arc length revealed significantly correlated with tear meniscus height in DED patients. **Conclusions**: DED and its severity can affect corneal biomechanics. Tear volume on the ocular surface could be one of the important factors to influence corneal biomechanics.

## 1. Introduction

Dry eye disease (DED) is a global issue related to tear film instability, ocular surface inflammation, and neurosensory dysfunction [1,2]. It can be marked by complex changes in the tear film and ocular surface, which further cause ocular discomfort and visual issues [2]. Mainstay treatments for DED include, but are not limited to, instilling artificial tears, improving eyelid hygiene, and making lifestyle and environmental modifications [3,4]. With its chronic nature and the need for ongoing management and treatment, DED may lead to long-term cost barriers worldwide and generate a substantial economic burden [5].

In the past decade, only few studies proposed that the disease entity of DED is linked to alterations in corneal biomechanics [6,7,8,9]. The possible mechanisms linking DED to changes in corneal surface characteristics may be attributed to insufficient maintenance of ocular epithelial surface due to decreased lubrication effects of tears [10]. This leads to chronic inflammation and disruption of meibomian glands, driven by the cytokine effects of T helper type 1 (Th1)-derived interferon (IFN)-γ and T helper type 17 (Th17)-derived interleukin (IL)-17 [11,12]. The Th17 pathway may specifically induce the expression of Matrix metalloproteinase (MMP)-3 and MMP-9, leading to corneal epithelial barrier injury and further worsening the severity of DED [12,13]. Previous limited clinical studies have reported that DED patients may have more compliant cornea, which could be related to changes in corneal biomechanics [8,9,10].

Common techniques for evaluating corneal biomechanics are based on the viscoelastic characteristics of the cornea [14]. These techniques, including applanation tonometry, Brillouin scattering, and elastography, are able to apply a well-defined stimulus to generate a subsequent visualized tissue response for quantification and analysis [15]. However, only the applanation tonometry-based device is currently available for clinical use. Corneal Visualization Scheimpflug Technology (Corvis ST; Oculus, Wetzlar, Germany) is a representative applanation tonometry device which provides state-of-the-art corneal biomechanic measurements. Corvis ST can obtain information on corneal biomechanics by applying a noncontact air puff with fixed force to the ocular surface and recording the time-series changes in corneal shape with an ultrahigh-speed Scheimpflug camera [16].

The impact of DED on corneal biomechanics has not yet been well investigated. Moreover, the relationship between tear film homeostasis and corneal biomechanics has not been clearly elucidated. Although some studies have suggested that tear film physiology, hydration status, and ocular surface microstructures may influence corneal biomechanics [17,18,19,20], clinical data directly linking DED to in vivo biomechanical changes remain limited. Hence, this study aimed to compare the corneal biomechanics of DED patients via a corneal biomechanical analyzer, Corvis ST. Furthermore, we tried to explore the association between tear film homeostatic indexes and corneal biomechanical parameters for DED patients with various severities.

## 2. Materials and Methods

### 2.1. Participants

This prospective cohort was conducted from October 2020 to September 2023 at Kaohsiung Chang Gung Memorial Hospital (KCGMH), Taiwan. The study adhered to the tenets of the Declaration of Helsinki and was approved by the Institutional Review Board of KCGMH. We included patients without and with DED and divided them into two groups: a control group (non-DED) and a DED group. DED patients were identified by the presence of positive dry eye symptoms [Ocular Surface Disease Index (OSDI) > 13] and at least one of the following two conditions: average non-invasive keratograph break-up time (NIKBUT-ave) ≤ 6 s or Oxford staining score (OSS) ≥ 1, in accordance with the refined global definition of DED proposed in the Tear Film and Ocular Surface Society Dry Eye WorkShop II (TFOS DEWS II) in 2017 [21]. All measurements were performed in a controlled environment with temperature 24 °C in the summer and 20 °C in the winter and with 55% relative humidity). Patients were instructed to refrain from instillation of artificial tears or excessive blinking for at least 5 min prior to testing.

Subjects were excluded if they were younger than 20 or older than 80 years, had an active ocular infection, or had ocular diseases such as corneal dystrophy, glaucoma, keratoconus, uveitis, or intraocular tumors. Further exclusion criteria included prior ocular or eyelid surgery within 6 months, contact lens use within 1 month before examination, pregnancy, and systemic conditions known to affect corneal biomechanics such as diabetes mellitus and autoimmune diseases. Patients unable to complete the examinations were also excluded.

All participants first completed the OSDI questionnaire to quantify their subjective ocular symptoms. Subsequently, they underwent an orderly series of ocular examinations, including measurement of the tear meniscus height (TMH), detection of the non-invasive tear break-up time, meibography, and corneal fluorescence staining. Finally, corneal biomechanics were analyzed with the Corvis ST. The right eye was chosen as the examined eye if both eyes met the enrolled criteria, while the left eye was measured when the right eye was not qualified.

### 2.2. Defining the DED and Non-DED

The enrolled subjects were divided into two groups based on having DED or not, which was defined by the values of average NIKBUT (NIKBUT-ave) [22,23,24]. The subjects with NIKBUT-ave < 6 s were classified into the dry eye group, while those with NIKBUT-ave > 6 s were grouped into the control group, as known as non-DED group.

### 2.3. Evaluating Dry Eye Symptoms

This OSDI questionnaire is a tool designed to assess the subjective severity of dry eye, which comprises 12 questions, scoring from 0 to 4 based on the symptoms, frequency, and the effect of these symptoms on vision-related functioning, with higher scores indicating more severe impairment [25]. The overall score is calculated with a scale of 0 to 100. Scores were then measured and reported for each individual upon completion.

### 2.4. Assessing the Tear Film Homeostasis

Keratograph^®^ 5M (K5M; Oculus, GmbH, Wetzlar, Germany) is a video topographer that can assess both the quantity and quality of the tear film automatically and objectively in a non-invasive manner [25]. TMHs were initially measured perpendicular to the central eyelid margin relative to the pupil center. NIKBUT can be measured by K5M between a blink and the defect shown on the rings reflected on the tear surface [26]. NIKBUT-1st, NIKBUT-ave, and NIKBUT tolerability (the tolerable time of the NIKBUT test) were then obtained at least 5 min after the TMH measurement. With a near-infrared illumination with 840 nm diode light source, meibography was performed to directly visualize the morphology of meibomian glands in vivo in a non-invasive manner finally. The severity of the meibomian gland dropout can be graded according to the meibograde, from degree 0 to 4, with each degree of rise representing a 25% meibomian gland dropout [27].

### 2.5. Examining Corneal Surface Injuries

OSS is a grading score for grading corneal surface damage in dry eye by the Oxford Schema [28]. After applying fluorescent dye to the eye, the stained cornea was examined under absorption filters. Staining ranges from 0 to 5 for the cornea. Moreover, the grade can be added if filaments, pupillary area staining, and confluent patches present while examining.

### 2.6. Determining Corneal Biomechanics

Corvis ST is a clinically available instrument that records the corneal response to a defined air pulse with high-speed Scheimpflug camera, which can capture over 4300 images per second, allowing direct measurement of intraocular pressure (IOP) independent to corneal thickness (biomechanically corrected IOP, bIOP) during first applanation (A1) as well as central corneal thickness. Physicians can also obtain other properties of corneal biomechanics during the phases of A1, highest concavity (HC), and the second applanation (A2) with this instrument. Moreover, some novel parameters can indicate ocular biomechanic limits (limit indices) and biomechanical correction parameters with physiological significance (integrated parameters) [29,30]. Figure S1 showed the representative Corvis ST evaluation for a non-DED (Figure S1a) and a (Figure S1b) DED patients.

### 2.7. Statistical Analysis and Power Calculation

The subjects’ characters and their ocular parameters for the control and DED groups were compared and statistically tested by the Fisher’s exact test or the Mann–Whitney U test. The association between the Corvis ST parameters and the tear film homeostatic parameters were analyzed via Spearman’s rank-order correlation. All of the data were analyzed using the SPSS software, version 22 (IBM Corp., Armonk, NY, USA). A priori sample size estimation was performed using G*Power 3.1, based on previously reported differences in NIKBUT-ave between DED and normal populations [18]. With an expected effect size (Cohen’s d) of approximately 1.3, a two-tailed test with α = 0.05 and power = 0.8 indicated that a minimum of 22 subjects (11 per group) would be sufficient.

For the above analyses, a two-tailed *p* value less than 0.05 was considered statistically significant.

## 3. Results

### 3.1. Population Characteristics

A total of 72 patients, including 34 patients with DED and 38 patients without DED, were compared and analyzed. Among the basic characters, there was no significant difference between the two groups (Table 1). For the tear film homeostatic parameters, the DED group had significantly shorter NIKBUT-1st, NIKBUT-ave, and tolerable time of the NIKBUT test than the control group (Table 1).

### 3.2. Comparison of Corneal Biomechanics Between the Non-DED and DED Patients

DED patients showed significantly different performance in some biomechanical parameters of A1, A2, and limit indices (Table 2). Among A1 indices, both A1 delta arc length (A1 dArcL) and A1 deflection area (A1 Darea) in the DED group showed significantly greater values (*p* < 0.05) than those in the control group. Among A2 indices, the DED group showed significantly higher A2 deformation amplitude (A2 DA) than the control group. Among the limit indices, DED patients had significantly greater whole eye movement (WEM) than non-DED patients (Table 2).

### 3.3. Relationships of Corvis ST Parameters and Dry Eye Parameters

**Figure 1:**
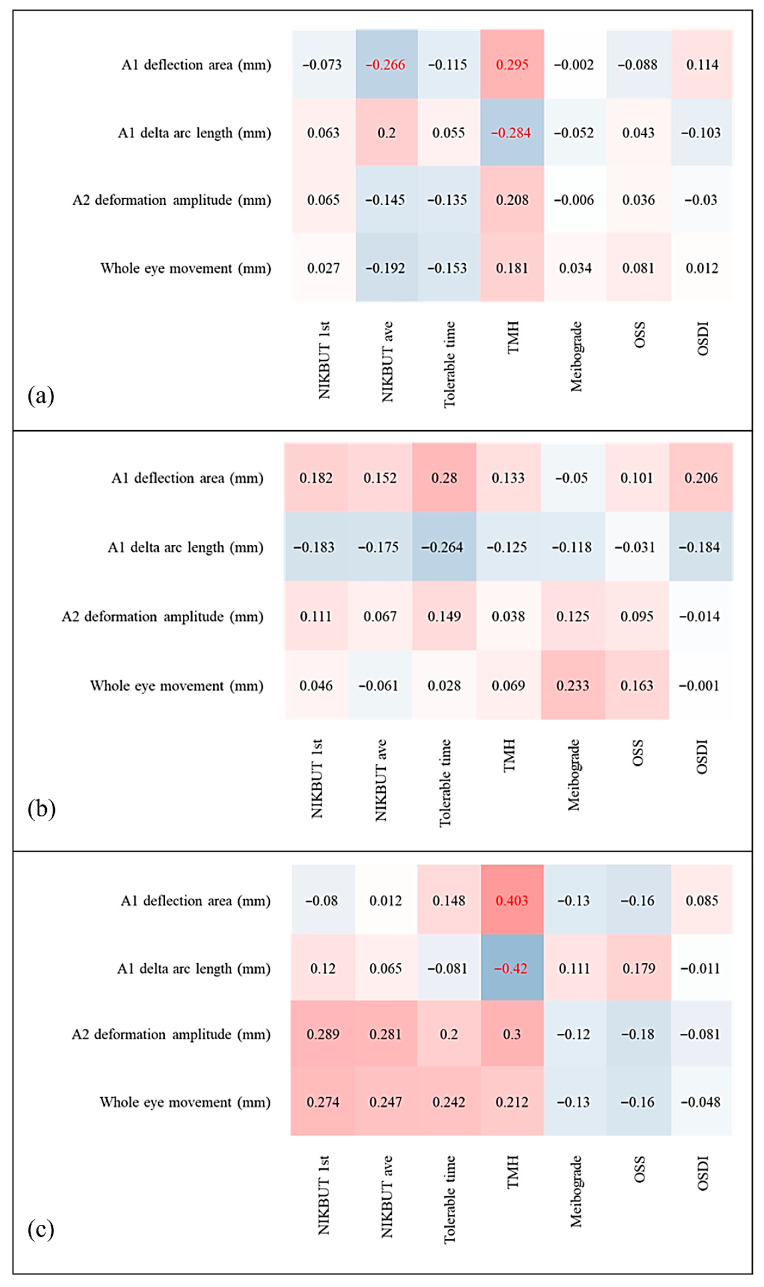
Correlation matrices between Corvis ST corneal biomechanical parameters and K5M parameters for (**a**) all patients, (**b**) non-DED subjects only, and (**c**) DED subjects only. Red columns show positive correlations, and blue columns show negative correlations; darker shades indicate stronger relationships. Red statistics indicate statistical significance.

**Figure 2:**
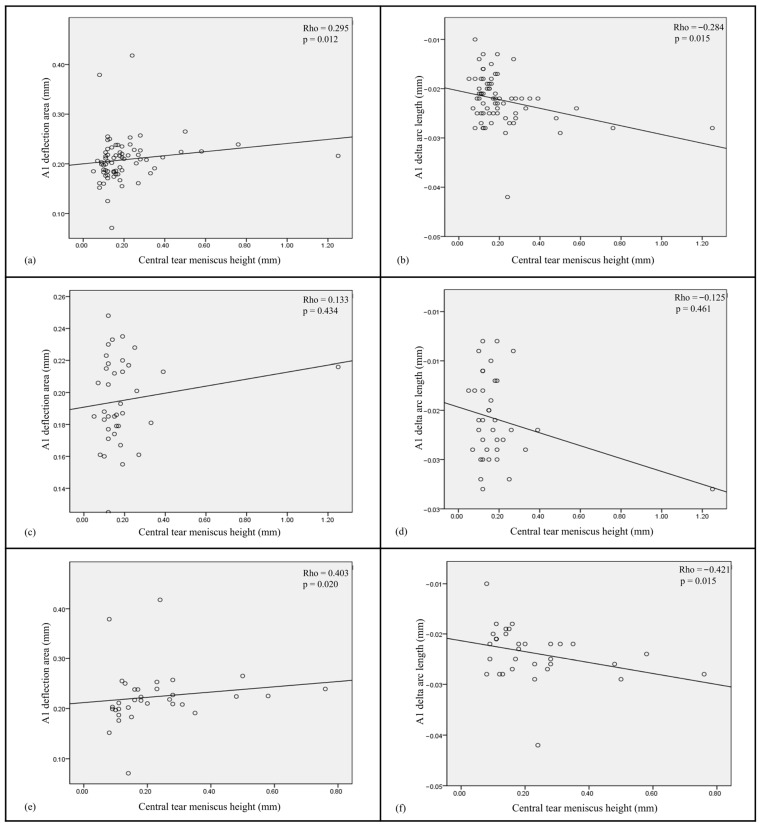
Scatter plots show correlations between two significant corneal biomechanical parameters (A1 deflection area and A1 delta arc length) and the tear meniscus height for (**a**,**b**) all subjects, (**c**,**d**) non-DED patients, and (**e**,**f**) DED subjects.

Dry eye parameters and biomechanical indices demonstrated a significant positive association between the A1 Darea and NIKBUT-ave in the overall population (Figure 1a). Furthermore, both the A1 Darea and A1 dArcL were found to be significantly positively and negatively correlated with central TMH (*p* = 0.012 and 0.015, respectively) (Figure 2a,b). In contrast, no significant correlations were observed between the Corvis indices and dry eye parameters within the control group (Figure 1b and Figure 2c,d). However, in the DED group, both the A1 Darea and A1 dArcL showed significant positive and negative associations with central TMH (*p* = 0.020 and 0.015, respectively) (Figure 1c and Figure 2e,f).

## 4. Discussion

This study explored the impact of DED on corneal biomechanics in DED patients. We found that all participants have distinct presentations in A1, A2, and limit corneal biomechanical indices, including A1 dArcL, A1 Darea, A2 DA, and WEM. Moreover, we examined the association between Corvis indices and dry eye parameters. In all subjects, the result showed positive correlations between A1 Darea and both NIKBUT-ave and central TMH. A1 dArcL was negatively associated with central TMH. In the dry eye group, similar findings were noted as well. However, no such correlations were observed in the control group.

In our study, DED patients had significantly higher A1 Darea, A1 dArcL, A2 DA, and WEM than non-DED patients (Table 2), which implied the former’s corneas were more compliant or had less stiffness. In addition, the severity of DED was found associated with the corneal biomechanics as well, with the more severe the DED was, the more compliant the cornea was (Figure 1c and Figure 2e,f). The A1 Darea is the area of the applanated region at the first applanation, which represents the corneal movement without whole eye motion and is the indicator of corneal elasticity. More compliant corneas are reported to have higher A1 Darea [31,32]. A1 dArcL means the delta arc length at the first applanation. Greater values of A1_dArcL have also been reported in keratoconus, post-laser in situ keratomileusis (LASIK), and post-LASIK keratectasia eyes [33]. DA represents the vertical movement of the central cornea and is related to corneal stiffness and the IOP. The stiffer the cornea is, the smaller the DA. WEM, on the other hand, is the vertical movement of the whole eye, and is affected by scleral and underlying soft tissue’s biomechanical properties.

Compared with previous studies focusing on corneal biomechanics in DED, their results were aligned with ours. Satitpitakul et al. reported that greater DED severity was associated with less stiffness of cornea in patients with either low or normal tear production [8]. They found significant associations between conjunctival staining scores and A2 velocity, between corneal staining scores and A2 length (A2L), and between Schirmer test with A1 time and A2L. Moreover, Long et al. found that dry eye patients had decreased HC time than normal subjects [9]. Furthermore, Yang et al. implied that ocular surface injuries in DED could be made due to increased mean eyelid pressure and increased shear stress, which may affect corneal cell behavior by altering mechanical factors [10].

There has been no previous study dedicated on the correlation between tear film homeostasis properties measured by K5M and the corneal biomechanism analyzed with Corvis ST. According to our analysis, the corneas of the DED patients were more compliant, which could be reflected by the positive association between A1 Darea and NIKBUT-ave as well as between A1 dArcL and NIKBUT-ave. A1 dArcL had a negative association with central TMH, which was partially consistent with the results by Tung et al. [34]. They found that tear volume may not completely reflect the DED severity. Instead, for patients with meibomian gland dysfunction, higher tear volume correlated with worse ocular surface disease due to change in tear osmolarity, tear compositions (e.g., lactoferrin concentration, inflammatory mediators), and delayed tear clearance, which could be related to corneal epithelial damage [34].

Our result showed that DA and WEM also had no significant associations with any of the tear film homeostasis parameters. The complex effects of corneal thickness, IOP, ocular structures, and orbital soft tissue may contribute to this result [35]. Different corneal biomechanical parameters reflect diverse ocular properties, not merely tear film status. DA reflects the maximal displacement of the cornea under load, which may be dominated by the stroma’s bulk elasticity and lamellar microstructure, buffering the more superficial influences of tear film instability. In addition, higher IOP and thicker corneas can lead to lower values of DA [36,37]. WEM may echo the biomechanical status of the sclera, orbital soft tissues, and boundary conditions, making them less sensitive to superficial tear film changes [38]. Thus, it is reasonable to note that not all corneal biomechanical parameters had significant associations with tear film homeostasis parameters.

Beyond alterations in tear volume, the physiology and pathology of the tear film itself are important contributors to corneal biomechanics. The tear film, composed of lipid, aqueous, and mucin layers, maintains ocular surface homeostasis, while its disruption can induce surface irregularities, neovascularization, and inflammation-driven remodeling of the extracellular matrix [17,20,39,40].

Experimental and imaging studies further demonstrate that tear adhesiveness, redistribution, and meniscus dynamics modulate corneal stress distribution [17], and that hydration critically governs lamellar spacing and collagen interactions, with environmental or eyelid factors inducing structural shifts [39]. At the microstructural level, stromal striae arranged in truss-like geometries enhance biomechanical resilience [40,41]. In dry eye disease, chronic tear film instability, epithelial barrier dysfunction, and sustained inflammation may disrupt these hydration-dependent and microarchitectural mechanisms, leading to increased corneal compliance. Additionally, unstable tear film can bias in vivo biomechanical measurements, further complicating interpretation [18,19,42,43]. Collectively, these considerations suggest that the biomechanical alterations observed in DED reflect both genuine structural remodeling and measurement variability mediated by tear film dynamics.

Currently, DED may be clinically diagnosed using various assessment tools, such as various dry eye questionnaires, different ocular surface staining grading systems, invasive or non-invasive tear break-up tests, tear secretion, tear volume, and meibography [44,45]. Among the above measurements, tear break-up time (TBUT) has been proposed to be one of the mostly used modalities since tear film instability is one of the leading pathophysiological features [23,46]. Moreover, NIKBUT can provide similar results to the conventionally invasive means [22,24], and may provide better repeatability and reproducibility in measuring DED due to less influence of reflex tearing during the examination [22,26,47].

In the present study, the classification of non-DED and DED groups were based on NIKBUT-ave threshold of 6 s as the cutoff value, which has been supported in multiple studies as a practical and objective tool for DED diagnosis [22,23,24]. The 2017 TFOS DEWS II report proposed a NIKBUT < 10 s as a general diagnostic criterion for dry eye, with ≤5 s being more specific for clinical disease. But this broad range includes patients with varying severity [44]. Subsequent studies have further illustrated the optimal cutoff duration in stratifying DED and non-DED patients. In a randomized controlled trial by Wang et al., NIKBUT median of 6.3 s was observed in symptomatic DED subjects, suggesting this value represents a clinically meaningful division between symptomatic DED and healthy groups [22]. In addition, for symptomatic DED patients, NIKBUT-ave were found 0.2–2 s longer than TBUT values [23,24], implying that NIKBUT-ave 6 s is one of the reasonable cutoffs for identifying DED patients.

In this study, there were several limitations. First of all, the effects of eyedrops were not analyzed. Most of these subjects were regularly followed up and treated by various lubricants. Despite this, all of the participants were asked to discontinue ointment one day before the examination and stop any eyedrops instillation on the examination day until completion of all tests. Therefore, we deemed that its effects may be relatively subtle in affecting the results. Second, the diagnostic criteria for DED were based on TFOS DEWS II since the patients were recruited before the publication of the latest TFOS DEWS III [48]. Future studies investigating DED patients’ corneal biomechanics based on the latest guideline were needed. Third, we simply use NIKBUT-ave 6 s as cutoff to divide the two groups, which may inevitably neglect the potential impact of other dry eye related parameters. However, TBUT is the most widely used parameter to identify and grade DED and can reflect the severity of DED in most circumstances and correlate to other common parameters [23,46], making it simple yet feasible way to identify these patients clinically. Despite that we had performed power analysis to determine the minimum number of cases required in our study, future studies with a relatively larger sample size are still needed to explore the corneal biomechanics in DED patients.

Our study provides a new clinical perspective on corneal biomechanics in patients with DED. We found that corneas in DED are more compliant, reflecting altered biomechanical properties. These findings have several clinical implications. The severity of DED may be assessed not only by traditional tear film parameters but also by evaluating corneal structural changes, which could be especially useful in the follow-up of patients at risk of corneal melting. Increased corneal compliance in DED may also bias IOP measurements and reduce resistance to mechanical stress, with potential consequences for refractive surgery and keratoplasty outcomes. Moreover, incorporating biomechanical indices with tear film assessments may enhance disease stratification and provide complementary markers for monitoring therapeutic response.

## 5. Conclusions

In conclusion, corneal biomechanics of DED patients could be different from non-DED subjects. In addition, the more severe the dry eye is, the more compliant the cornea is. Moreover, tear film stability reflected by the tear break-up time may be one of the most crucial factors associated with corneal biomechanics in eyes of DED. For DED patients, especially for those with higher severity, stabilizing the tear film could improve their corneal biomechanics.

## Figures and Tables

**Table 1:** Demographic data for subjects of non-DED and DED.

Characteristics *^a^*	All Participants (*n* = 72)	Non-DED (*n* = 38)	DED (*n* = 34)	*p* Value
Basic characters				
Age (year)	64.0 (15.0)	62.0 (12.0)	64.0 (17.0)	0.458
Gender (female; %)	71 (92.2)	37 (97.4)	31 (91.2)	0.338
Sjogren syndrome (%)	40 (55.6)	19 (50)	21 (61.8)	0.350
SE (diopter)	−0.25 (3.75)	−0.25 (3.88)	−0.13 (3.37)	0.721
IOP (mmHg)	15.0 (3.5)	15.0 (3.6)	14.5 (3.1)	0.874
Tear film homeostatic parameters				
OSDI	49.0 (67.8)	48.0 (34.5)	50.0 (47.5)	0.491
NIKBUT-1st (s)	4.01 (2.47)	4.40 (3.15)	3.44 (1.80)	0.001 *
NIKBUT-ave (s)	6.14 (3.87)	8.48 (7.34)	4.59 (2.47)	<0.001 *
NIKBUT tolerability (s)	10.13 (7.20)	12.78 (12.83)	6.18 (3.20)	<0.001 *
TMH (mm)	0.16 (0.13)	0.15 (0.07)	0.17 (0.17)	0.255
Meibograde	2.0 (2.0)	2.0 (2.0)	3.0 (2.0)	0.396
OSS	0.0 (1.0)	0.0 (1.0)	0.5 (1.8)	0.278

*^a^* Continuous data were presented as “median  ± interquartile range”, and categorical data were presented as “*n* (%)”. Mann–Whitney U test was used for analysis; gender and Sjogren syndrome were analyzed with Fisher’s exact test. *p* < 0.05 was recognized as a significant difference in statistics (*). SE = spherical equivalence; IOP = intraocular pressure; OSDI = ocular surface disease index; NIKBUT-1st = the first non-invasive keratograph break-up time; NIKBUT-ave = the average non-invasive keratograph break-up time; NIKBUT tolerability = the tolerable time of the NIKBUT test; TMH = tear meniscus height; OSS = Oxford staining score, i.e., corneal surface staining grade based on the Oxford scheme; DED = dry eye disease.

**Table 2:** Comparison of Corvis ST parameters between the non-DED and DED patients.

Corvis ST Parameters *^a^*	Non-DED (*n* = 38)	DED (*n* = 34)	*p* Value *^b^*
Basic parameters			
A1 indices			
A1 Time (ms)	7.12 (0.52)	7.09 (0.41)	0.897
A1 Velocity (m/s)	0.16 (0.03)	0.16 (0.03)	0.685
A1 DA (mm)	0.15 (0.02)	0.15 (0.02)	0.693
A1 DA_c (mm)	0.11 (0.01)	0.11 (0.01)	0.063
A1 DL (mm)	2.41 (0.28)	2.47 (0.22)	0.092
A1 dArcL (mm)	−0.022 (0.007)	−0.023 (0.006)	0.024 *
A1 Darea (mm^2^)	0.19 (0.04)	0.22 (0.04)	0.002 *
A2 indices			
A2 Time (ms)	20.6 (0.5)	20.7 (0.4)	0.875
A2 Velocity (m/s)	−0.39 (0.06)	−0.39 (0.05)	0.664
A2 DA (mm)	0.41 (0.11)	0.46 (0.12)	0.024 *
A2 DA_c (mm)	0.13 (0.03)	0.13 (0.02)	0.477
A2 DL (mm)	2.81 (0.93)	2.96 (0.89)	0.531
A2 dArcL (mm)	−0.03 (0.01)	−0.03 (0.01)	0.326
A2 Darea (mm^2^)	0.28 (0.11)	0.30 (0.10)	0.463
HC indices			
HC Time (ms)	17.3 (0.8)	17.3 (0.9)	0.772
HC DA (mm) *^c^*	1.12 (0.17)	1.12 (0.17)	0.644
HC DA_c (mm)	0.92 (0.20)	0.90 (0.17)	0.835
HC DL (mm)	6.29 (0.84)	6.23 (0.78)	0.681
HC dArcL (mm)	−0.15 (0.03)	−0.15 (0.03)	0.580
HC Darea (mm^2^)	3.17 (0.98)	3.16 (0.83)	0.875
Limit indices			
WEM (mm)	0.30 (0.14)	0.36 (0.13)	0.021 *
WEM time (ms)	21.6 (1.4)	21.9 (0.7)	0.183
Max DA (mm) *^c^*	1.12 (0.17)	1.12 (0.17)	0.644
Max DA_c (mm)	0.95 (0.17)	0.92 (0.12)	0.616
Max DA_c time (ms)	15.7 (1.7)	16.4 (1.5)	0.191
Max dArcL (mm)	−0.17 (0.05)	−0.19 (0.04)	0.112
Max ICR (mm^−1^)	0.19 (0.03)	0.19 (0.02)	0.826
Peak Distance (mm)	4.96 (0.46)	4.81 (0.36)	0.652
Radius (mm)	6.47 (1.20)	6.75 (1.20)	0.787
Integrated parameters			
bIOP (mmHg)	14.3 (3.5)	13.6 (2.5)	0.202
SSI (mmHg/mm)	1.18 (0.34)	1.26 (0.39)	0.299
Pachy Slope (a.u.)	41.3 (18.6)	45.8 (12.2)	0.143
Max DA Ratio 1 mm (a.u.)	1.57 (0.09)	1.57 (0.06)	0.588
Max DA Ratio 2 mm (a.u.)	4.48 (0.84)	4.38 (0.59)	0.830
ARTh (mm)	490 (184)	482(166)	0.443
Integrated Radius (mm)	8.42 (1.34)	8.17 (1.22)	0.600
SP-A1 (mmHg/mm)	99.5 (24.2)	95.2 (23.9)	0.398
CBI (a.u.)	0.36 (0.44)	0.31 (0.48)	0.795

*^a^* All variants were shown in median  ± interquartile range; *^b^* statistical test by the Mann–Whitney U test; *p* < 0.05 was recognized significant difference in statistics (*). *p* < 0.10 was highlighted in bold. *^c^* Max DA = HC DA. DED = dry eye disease; A1 = the first applanation; DA = deformation amplitude; DA_c = deflection amplitude; DL = deflection length; dArcL = the change of arc length within 7 mm of corneal center; Darea = deflection area; A2 = the second applanation; HC = the highest concavity; WEM = whole eye movement; Max = maximal; ICR = inverse concave radius; bIOP = biomechanically corrected intraocular pressure; SSI = stress strain index; Pachy Slope (a.u.) = the slope of corneal thickness (arbitrary unit); DA Ratio 1 mm (2 mm) = DA ratio between cornea apex and paracentral 1 mm (2 mm); ARTh = Ambrosio Relational Thickness horizontal; SP-A1 = stiffness parameter at the first applanation; CBI = corneal (or Corvis) biomechanical index.

## Data Availability

Data can be made available by the authors upon reasonable request.

## Supplementary Materials

The following supporting information can be downloaded at https://www.mdpi.com/article/10.3390/biomedicines13102524/s1. Figure S1: Representative Corvis ST evaluation for all participants. a. A non-DED patient. b. A DED patient shows more compliant cornea than a non-DED patient.

## Abbreviations

The following abbreviations are used in this manuscript:

DED	Dry eye disease
Corvis ST	Corneal Visualization Scheimpflug Technology
K5M	Keratograph^®^ 5M
IOP	Intraocular pressure
SE	Spherical equivalence
OSDI	Ocular surface disease index
NIKBUT-1st	The first non-invasive keratograph break-up time
NIKBUT-ave	The average non-invasive keratograph break-up time
TMH	Tear meniscus height
OSS	Oxford staining score
A1	The first applanation
A2	The second applanation
DA	Deformation amplitude
DA_c	Deflection amplitude
DL	Deflection length
dArcL	The change of arc length within 7 mm corneal center
Darea	Deflection area
HC	The highest concavity
WEM	Whole eye movement
Max	Maximal
ICR	Inverse concave radius
bIOP	Biomechanically corrected intraocular pressure
SSI	Stress strain index
Pachy Slope (a.u.)	The slope of corneal thickness (arbitrary unit)
DA Ratio 1 mm (2 mm)	DA ratio between cornea apex and paracentral 1 mm (2 mm)
ARTh	Ambrosio Relational Thickness horizontal
SP-A1	Stiffness parameter at the first applanation
CBI	Corneal (or Corvis) biomechanical index
