# Supplementary material for: The Impact of Dry Eye Disease on Corneal Biomechanics Analyzed with Corneal Visualization Scheimpflug Technology
Source: Biomedicines. 2025 Oct 16;13(10):2524. doi: 10.3390/biomedicines13102524 (PMC12561406; doi:10.3390/biomedicines13102524)
Supplement: Supplementary file 1 [file biomedicines-13-02524-s001.zip › biomedicines-3873001-supplementary.pdf]

Supplemental Materials

The Impact of Dry Eye Disease on Corneal Biomechanics Analyzed with Corneal Visualization Scheimpflug Technology

Li-Wen Chiu, Ren-Wen Ho, Hun-Ju Yu, Po-Chiung Fang, I-Hui Yang, Ming-Tse Kuo

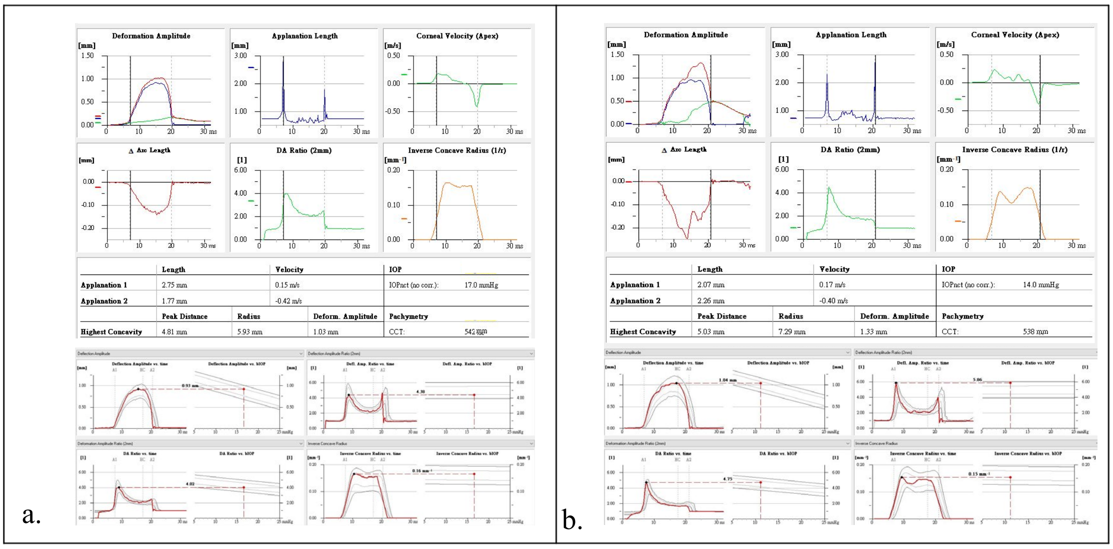

Figure S1. Representative Corvis ST evaluation for all participants. a. A non-DED patient. b. A DED patient shows more compliant cornea than a non-DED patient.
